# New low-dose liquid pilocarpine formulation for treating dry mouth in Sjögren’s syndrome: clinical efficacy, symptom relief, and improvement in quality of life

**DOI:** 10.1186/s40780-018-0099-x

**Published:** 2018-03-01

**Authors:** Machiko Watanabe, Chisato Yamada, Yoshinori Komagata, Hirotoshi Kikuchi, Hiroyuki Hosono, Fumio Itagaki

**Affiliations:** 1 Laboratory of Clinical Pharmaceutics, Faculty of Pharma-Science, Teikyo University, 2-11-1 Kaga, Itabashi-ku, Tokyo, 173-8605 Japan; 2 Department of Internal Medicine, Teikyo University School of Medicine, 2-11-1 Kaga, Itabashi-ku, Tokyo, 173-8605 Japan; 3 First Department of Internal Medicine, Kyorin University School of Medicine, 6-20-2 Shinkawa, Mitaka-shi, Tokyo, 181-8611 Japan

**Keywords:** Sjögren’s syndrome, Pilocarpine, Xerostomia, Dry mouth, Sodium alginate

## Abstract

**Background:**

Patients with Sjögren’s syndrome (SS) typically present clinically with xerostomia (dry mouth) because of progressive damage to the exocrine glands. We developed a new, low-dose pilocarpine/sodium alginate (LPA) solution with pilocarpine hydrochloride to inhibit systemic adverse effects by administering via the oral mucosa. The purpose of this study was to assess its stability, safety, and efficacy.

**Methods:**

The pilocarpine concentration in an LPA liquid formulation was measured 3, 7, 14, and 28 days after preparation to assess its stability. A prospective clinical trial was undertaken to assess the efficacy and safety of the LPA solution as a symptomatic treatment for dry mouth in SS. Patients (*n* = 24) with clinically significant xerostomia were enrolled after providing written informed consent. Whole-mouth salivary flow rate was measured twice; immediately before and 60 min after LPA application. Symptoms were assessed by questionnaire with visual analog scales or checkboxes before the first application (baseline), and then once daily for 7 days.

**Results:**

The pilocarpine content 3, 7, 14, and 28 days after preparation showed no marked change, confirming its stability. Salivary flow was significantly increased from 0.076 ± 0.092 g/30 s to 0.122 ± 0.140 g/30 s 60 min after LPA administration (*P* < 0.001). Dry mouth and thirstiness showed significant improvement compared with that of baseline (*P* ≤ 0.01). The only adverse effect was sweating, and no serious drug-related adverse events were reported.

**Conclusions:**

This new, low-dose pilocarpine formulation was well-tolerated and resulted in significant improvements in symptoms of dry mouth and other xerostomic conditions in patients with SS.

**Trial registration:**

The study approval number in the institution; 08–068-2. Registered January 19, 2009. UMIN000029307. Registered 27 September 2017 (retrospectively registered).

## Background

Sjögren’s syndrome (SS), a chronic autoimmune disease with slow progression, is characterized by lymphocytic infiltration into the exocrine glands, resulting in xerostomia. As a result of exocrine dysfunction, most patients with SS have symptoms related to diminished activity of the salivary glands, which can cause difficulty chewing and swallowing food and speaking without frequent water intake [1]. Moreover, the chronicity of the disease can lead to significant debility and decreased patient quality of life [2, 3].

Pilocarpine is a parasympathomimetic agent that functions primarily as a muscarinic agonist with mild β-adrenergic activity. This alkaloid causes pharmacologic stimulation of exocrine glands in humans, resulting in diaphoresis, salivation, lacrimation, and gastric and pancreatic secretion. Although pilocarpine hydrochloride tablets are currently indicated for the treatment of xerostomia in SS, their toxicity is frequently reported [4, 5].

We developed a new, low-dose pilocarpine formulation of a pilocarpine/sodium alginate (LPA) solution. In previous reports, we demonstrated that sodium alginate (Alg-Na) is effective for retention of moisture in the mouth of patients with xerostomia [6, 7]. We, therefore, hypothesized that Alg-Na would be helpful in alleviating symptoms of xerostomia and would provide a useful base for the pilocarpine formulation. As a medium, Alg-Na was used in the low-dose pilocarpine formulation to inhibit systemic adverse effects.

After investigating the stability of the LPA solution, a prospective clinical trial was undertaken to assess its efficacy and safety as a symptomatic treatment for dry mouth in SS. Herein, we present the results of that clinical trial.

## Methods

### Preparation

Pilocarpine, Alg-Na, and sodium bicarbonate (NaHCO_3_) were purchased from Wako Pure Chemical Industries (Osaka, Japan). LPA solution (0.3% pilocarpine, 1% Alg-Na, and 0.1% NaHCO_3_, *w*/*v*) was prepared as follows. Pilocarpine was completely dissolved in 0.1% (w/v) paraben solution, and 1.0 g Alg-Na was added. Citric acid monohydrate (0.12 g), 1.1 g trisodium citrate dihydrate, and 0.1 g NaHCO_3_ were added to make a final volume of 100 mL (pH 6.2). The LPA solution was packaged in Hybripack® tubes (0.32 mL, pilocarpine 0.96 mg). The dose volume was determined by estimating a single, well-spread application to the entire oral mucosa.

### Stability

Preparations were placed in light-blocking bags and stored at 4 °C. Pilocarpine content was determined 0, 3, 7, 14, and 28 days after preparation by high-performance liquid chromatography (HPLC) using an absolute calibration curve method. Samples were diluted 5000-fold with Milli-Q® water before being applied to the HPLC column. HPLC conditions, with a minor change from previous reports [8, 9], were as follows: column-oven temperature, 50 °C; mobile phase, 0.05 M potassium dihydrogen phosphate/methanol (95/5, *v*/v); and mobile phase flow rate, 1.0 mL/min. The eluate from the HPLC column was monitored for pilocarpine using an ultraviolet detector (λ = 214 nm).

### Patients

Approval was granted by the Teikyo University School of Medicine Ethics Committee. After the purpose and procedures of the study were explained in writing, written informed consent was obtained from all participants. Female SS patients (*n* = 24) with dry mouth (mean age, 61.8 ± 13.4 years) who visited the Internal Medicine Department of Teikyo University Hospital were enrolled. Their characteristics are shown in Table 1.

**Table 1:** Characteristics of patients with Sjӧgren’s syndrome and dry mouth

Number of patients (male / female)	24 (0/24)
Average age (years) ^a^	61.8 ± 13.4
Salivary secretion (g/30 s.) ^a^	0.076 ± 0.092
Major complications (cases)	
Osteoporosis	10
Rheumatism	7
Hypertension	7
Refractory gastroesophageal reflux disease	6
Keratitis	6
Diabetic	5
Hyperlipidemia	5
Systemic scleroderma	4
Insomnia	4
Clinical laboratory tests	
Anti-SS-A ^b^ antibody (cases)	
> 5 U/mL	17
< 5 U/mL	7
IgG (cases)	
< 800 mg/dL	2
800–1700 mg/dL	9
> 1700 mg/dL	11
Serum creatinine (mg/dL) ^a^	0.75 ± 0.24
Blood urea nitrogen (mg/dL) ^a^	14.6 ± 4.6
Aspartate aminotransferase (U/L) ^a^	23 ± 11
Alanine aminotransferase (U/L) ^a^	17 ± 7

^a^: Where indicated, data are expressed as means ± SD

^b^: Anti-Sjögren’s syndrome-related antigen A

### Assessment

A 10-cm visual analog scale (VAS; from “not at all uncomfortable” to “unbearably uncomfortable”) was used to assess the following five items representing intraoral conditions: “dryness”, “thirstiness”, “stickiness”, “altered taste”, and “painful tongue”. A 5-cm VAS was used to assess six items representing the following aspects of quality of everyday life: “general activities”, “normal work”, “interpersonal relationships”, “food intake”, “sleep” (from “sleep very well” to “cannot sleep at all”), and “appetite” (from “very good” to “completely diminished”). The 5-cm VAS ranged from “having no difficulty” to “having great difficulty”, unless otherwise specified. A 5-point scale (“better”, “slightly better”, “unchanged”, “slightly worse”, and “worse”) was used to assess overall changes in symptoms. Salivary flow was evaluated by measuring the amount of saliva absorbed into cotton rolls in 30 s.

### Treatment protocol

Prior to administering the LPA liquid formulation, participants were assessed for intraoral conditions, quality of everyday life, and salivary flow. The following instructions were given to participants before the first dose: 1) avoid swallowing and spread the liquid formulation thoroughly over the oral mucosa; 2) spit any remaining liquid 5 min after administration; and 3) avoid eating, drinking, and rinsing the mouth for 60 min after administration, or until salivary flow has been measured. For continuous LPA administration, participants were instructed to use one tube three times daily with doses 4 h apart for seven days and to follow instructions 1) and 2) above. Participants were asked to complete the assessment for changes in symptoms, intraoral conditions, side effects, and everyday life after completing the seven-day LPA course or terminating the course for any reason.

### Saliva collection

We used cotton rolls with a 1-cm diameter and a standardized 3-cm major axis. We first measured the weight of the dry cotton rolls, and then subtracted this from the weight of the cotton rolls soaked with saliva produced after 30 s.

### Statistical analysis

Salivary secretion (salivary flow) results are expressed as means, while the results of VAS assessment are expressed as median scores (1st and 3rd quartiles). Scores for intraoral conditions were analyzed by Scheffe’s F test; saliva flow measurements and scores for quality of everyday life were assessed using the Wilcoxon signed-rank test. Differences were deemed statistically significant at *P* < 0.05.

## Results

### Stability

The pilocarpine concentration in the LPA liquid formulation 3, 7, 14, and 28 days after preparation was 92.8%, 105.3%, 98.4%, and 98.6% of that measured immediately after preparation at baseline (Table 2), indicating no marked change in pilocarpine content for at least 4 weeks.

**Table 2:** Stability test of pilocarpine solution filled in a Hybripack® tube

Time (day)	Pilocarpine concentration (μg/mL) ^a^	Residual (%)
0	0.56 ± 0.03	100
3	0.52 ± 0.02	92.8
7	0.59 ± 0.01	105.3
14	0.55 ± 0.02	98.4
28	0.55 ± 0.01	98.6

^a^: Data are expressed as means ± SD (*n* = 3)

### Saliva collection

**Fig. 1:**
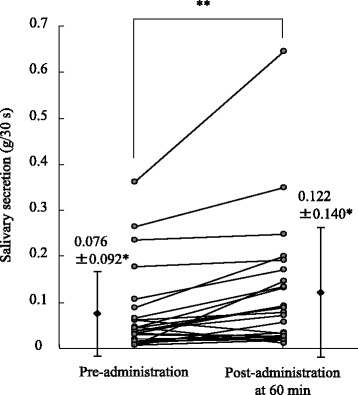
Change in salivary secretion by administration of pilocarpine solution to patients with Sjӧgren’s syndrome. *:Mean ± SD, *n* = 24; **:*P* < 0.01 (Wilcoxon signed-rank test)

**Fig. 2:**
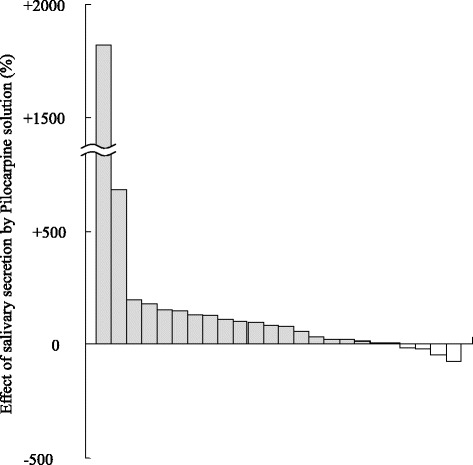
Salivary secretion after administration of pilocarpine solution to individual patients with Sjӧgren’s syndrome. Average 163 ± 381%, *n* = 24

The amount of saliva secreted 60 min after LPA administration (0.122 ± 0.140 g/30 s) was significantly higher than that measured before LPA administration (0.076 ± 0.092 g/30 s) (*P* < 0.01) (Fig. 1). Twenty participants (83%) showed elevated salivary secretion after LPA administration, albeit with noticeable individual variation in increments (Fig. 2).

### Assessment

As shown in Table 3, the median VAS scores for “dryness”, “thirstiness”, and “stickiness in the mouth” were > 5 before LPA administration, and these were reduced 60 min after the first dose of LPA and at the end of the 7-day LPA course (i.e., continuous LPA administration). In particular, post-administration (i.e., after the first dose and continuous administration) scores for “dryness” and “thirstiness” were significantly lower than the pre-administration scores (*P* < 0.05), suggesting good improvement.

**Table 3:** Self-reported oral effects of administration of pilocarpine solution to patients with Sjӧgren’s syndrome

Item		VAS (10 cm) ^b^	
	Pre-administration	Post-administration at 60 min	Continuous administration
Dryness	7.1 (6.1, 8.0)	4.4 (2.2, 6.5) ^a^	3.7 (2.0, 6.9) ^a^
Thirstiness	6.3 (4.5, 7.9)	2.3 (0.7, 5.4) ^a^	2.4 (1.5, 5.3) ^a^
Stickiness	6.3 (4.8, 7.4)	4.0 (0.7, 5.4)	2.4 (0.9, 6.5)
Altered taste	0.5 (0.3, 4.3)	1.1 (0.5, 4.3)	1.4 (0.5, 3.2)
Painful tongue	4.1 (0.4, 7.8)	1.3 (0.5, 6.4)	2.2 (0.4, 7.0)

^a^: Significantly different from pre-administration at *P* < 0.05 (Scheffe’s F test) (*n* = 21)

^b^VAS, visual analog scale; Median (1st quartile, 3rd quartile)

Median VAS scores for all items representing quality of everyday life, except “appetite”, were reduced after continuous LPA administration, indicating improvement following the treatment (Table 4). There were significant differences between pre- and post-administration scores for “general activities” (*P* < 0.01), “interpersonal relationships” (*P* < 0.01), and “food intake” (*P* < 0.05).

**Table 4:** Effect on daily activities following administration of pilocarpine solution to patients with Sjӧgren’s syndrome

Item	VAS (5 cm) ^a^	
	Pre-administration	Continuous administration
General activities (*n* = 21)	2.4 (1.2, 3.3)	1.0 (0.3, 2.0)**
Normal work (*n* = 21)	2.4 (1.0, 3.4)	1.0 (0.4, 2.1)
Interpersonal relationships (*n* = 20)	2.4 (1.5, 3.2)	1.4 (0.7, 2.4)**
Food intake (*n* = 18)	2.6 (1.0, 4.0)	1.4 (0.7, 3.0)*
Sleep (*n* = 20)	2.4 (0.8, 2.7)	1.5 (0.7, 2.5)
Appetite (*n* = 20)	0.9 (0.6, 2.4)	1.1 (0.5, 1.7)

*: Significantly different from pre-administration at *P* < 0.05; **: *P* < 0.01 (Wilcoxon signed-rank test)

^a^VAS, visual analog scale; Median (1st quartile, 3rd quartile)

All median scores for items representing experience after LPA administration were < 2.5; in particular, those for “taste”, “smell”, “irritation”, and “touch” were low (< 1.0) (Table 5).

**Table 5:** Effect on experiences following administration of pilocarpine solution to patients with Sjӧgren’s syndrome

Item	VAS (5 cm) ^a^	
	Pre-administration	Continuous administration
Taste	0.6 (0.2, 1.7)	0.9 (0.3, 2.6)
Smell	0.3 (0.1, 0.6)	0.4 (0.2, 1.3)
Irritation	0.4 (0.1, 0.6)	0.4 (0.2, 1.3)
Touch	0.4 (0.1, 1.7)	0.4 (0.2, 1.2)
Ease of use	2.4 (0.7, 3.5)	1.3 (0.8, 3.1)

^a^VAS, visual analog scale; Median (1st quartile, 3rd quartile), (*n* = 21)

Dry mouth symptoms were “better”, “slightly better”, and “unchanged” 60 min after the first dose of the LPA liquid formulation in 1, 17, and 5 participants, respectively, while symptoms were “worse” in 1 participant (Table 6). After continuous LPA administration, dry mouth symptoms were “better”, “slightly better”, and “unchanged” in 2, 8, and 9 participants, respectively (Table 6). One participant experienced slight hot flushes and flares after the initial dose and declined continuous LPA administration. Four participants experienced slight headache, dizziness, and hot flushes during continuous LPA administration.

**Table 6:** Effect on symptoms following administration of pilocarpine solution to patients with Sjӧgren’s syndrome

	Better	Slightly better	Unchanged	Slightly worse	Worse
Post-administration at 60 min*	1 (4.2%)	17 (70.8%)	5 (20.8%)	0 (0%)	1 (4.2%)
Continuous administration**	2 (10.5%)	8 (42.1%)	9 (47.4%)	0 (0%)	0 (0%)

*: *n* = 24; **: *n* = 19; values shown are number of individuals (percentage of total)

## Discussion

The pilocarpine content in the LPA liquid formulation was largely unchanged for 28 days after preparation in the dark at 4 °C, indicating that the formulation is stable when stored in the test container under the test conditions. On the basis of these findings, each dose was individually packed in a Hybripack® tube, and the expiration date under the specified conditions was labeled as “28 days after preparation in dark and cool conditions”.

The amount of saliva secreted increased significantly 60 min after LPA administration in SS patients with dry mouth. In addition, intraoral conditions and quality of everyday life were generally improved by continuous LPA administration. In particular, pre-administration and post-administration VAS scores for “dryness”, “thirstiness”, “general activities”, “interpersonal relationships”, and “food intake” differed significantly. These findings suggest that the increase in saliva secretion relieves dry mouth symptoms and alleviates conversation and eating difficulties, thereby improving the overall quality of everyday life. Furthermore, the 1% Alg-Na content appeared to be effective for relieving dry mouth symptoms and for achieving favorable retention and spread of the formulation thoroughly within the oral cavity.

It has been reported that oral pilocarpine mouthwash, which can be used at lower doses than tablets with reductions in some adverse effects, was not superior to that of 0.9% saline at relieving subjective oral dryness [10–12]. While a high incidence of hyperhidrosis (40.6%) is problematic in patients taking Salagen® tablets, the incidence in the present study was low (1 participant, 4.2%). The following additional adverse events, albeit mild, were caused by the LPA formulation: hot flushes (*n* = 3), as well as headache, dizziness, abdominal pain, and rhinitis (*n* = 1 each). Such adverse events are known in patients taking Salagen® tablets.

This study contains limitations. First, we conducted the study using an open-label design with a small number of cases, which might have led to subjective bias. Especially, the self-reported data of the patients may have been affected by the design. Second, although we estimated the effectiveness of pilocarpine solution before and after administration, this study was a single-arm design. Nevertheless, taken together, the new LPA formulation (pilocarpine content, 0.96 mg) is potentially a superior alternative to Salagen® tablets (pilocarpine content, 5 mg) for treating SS patients with dry mouth, and, in turn, improving their quality of life.

## Conclusions

The new LPA formulation improved intraoral xerostomic conditions and quality of everyday life in SS patients with dry mouth through increasing saliva secretion.

## Abbreviations

Alg-Na: Sodium alginate
HPLC: High-performance liquid chromatography
LPA: Low-dose pilocarpine/sodium alginate
SS: Sjögren’s syndrome
VAS: Visual analog scale
